# Accuracy of Step Count Estimations in Parkinson’s Disease Can Be Predicted Using Ambulatory Monitoring

**DOI:** 10.3389/fnagi.2022.904895

**Published:** 2022-06-16

**Authors:** Navid Shokouhi, Hamid Khodakarami, Chathurini Fernando, Sarah Osborn, Malcolm Horne

**Affiliations:** ^1^Global Kinetics Pty Ltd., Melbourne, VIC, Australia; ^2^Parkinson’s Laboratory, Florey Institute of Neurosciences and Mental Health, Parkville, VIC, Australia; ^3^Department of Clinical Neurosciences, St Vincent’s Hospital, Fitzroy, VIC, Australia

**Keywords:** step count, sensors, Parkinson’s disease, freezing of gait, gait, balance

## Abstract

**Objectives:**

There are concerns regarding the accuracy of step count in Parkinson’s disease (PD) when wearable sensors are used. In this study, it was predicted that providing the normal rhythmicity of walking was maintained, the autocorrelation function used to measure step count would provide relatively low errors in step count.

**Materials and Methods:**

A total of 21 normal walkers (10 without PD) and 27 abnormal walkers were videoed while wearing a sensor [Parkinson’s KinetiGraph (PKG)]. Median step count error rates were observed to be <3% in normal walkers but ≥3% in abnormal walkers. The simultaneous accelerometry data and data from a 6-day PKG were examined and revealed that the 5th percentile of the spectral entropy distribution, among 10-s walking epochs (obtained separately), predicted whether subjects had low error rate on step count with reference to the manual step count from the video recording. Subjects with low error rates had lower Movement Disorder Society Unified Parkinson’s Disease Rating Scale (MDS-UPDRS III) scores and UPDRS III Q10–14 scores than the high error rate counterparts who also had high freezing of gait scores (i.e., freezing of gait questionnaire).

**Results:**

Periods when walking occurred were identified in a 6-day PKG from 190 non-PD subjects aged over 60, and 155 people with PD were examined and the 5th percentile of the spectral entropy distribution, among 10-s walking epochs, was extracted. A total of 84% of controls and 72% of people with PD had low predicted error rates. People with PD with low bradykinesia scores (measured by the PKG) had step counts similar to controls, whereas those with high bradykinesia scores had step counts similar to those with high error rates. On subsequent PKGs, step counts increased when bradykinesia was reduced by treatment and decreased when bradykinesia increased. Among both control and people with PD, low error rates were associated with those who spent considerable time making walks of more than 1-min duration.

**Conclusion:**

Using a measure of the loss of rhythmicity in walking appears to be a useful method for detecting the likelihood of error in step count. Bradykinesia in subjects with low predicted error in their step count is related to overall step count but when the predicted error is high, the step count should be assessed with caution.

## Introduction

### Background

Altered gait characteristics occur early in Parkinson’s disease (PD) (Rehman et al., 2019) and even years prior to diagnosis (Del Din et al., 2019), with the most consistent abnormalities being a slower gait, increased variability, and asymmetry (Del Din et al., 2019; Rehman et al., 2019; Corra et al., 2021). These features are related to bradykinesia and improve with levodopa (Chien et al., 2006; Bryant et al., 2016; Corra et al., 2021). Approximately 25% of people with PD (PwP) have unstable posture at diagnosis (Kohat et al., 2021), and the incidence increases with time from diagnosis (Kohat et al., 2021) often with the development of freezing of gait (FOG) (Ge et al., 2020). This is associated with a shorter stride length (Nanhoe-Mahabier et al., 2011; Orcioli-Silva et al., 2018). These changes have been linked to cognitive dysfunction and anxiety (Giladi and Hausdorff, 2006; Weiss et al., 2015; Kueper et al., 2017; Yao et al., 2017). Executive dysfunction, revealed by dual tasking, affects the gait of PwP more than non-PD subjects (Salazar et al., 2017).

Although most studies of gait in PD have been conducted in laboratories, there has been increasing interest in ambulatory monitoring using sensors (Weiss et al., 2015) mostly because behavior in one’s natural environment is likely to differ from the formal environment of the laboratory (Robles-Garcia et al., 2015). Gait scores, particularly step count, have been assessed (Lamont et al., 2018; Straiton et al., 2018; Lai et al., 2020; Svarre et al., 2020), and while error of ≤3% in step count can be obtained with ambulatory sensors at normal walking speeds, error rate increases when walking is slower or higher than normal (Svarre et al., 2020) or discontinuous (Wendel et al., 2018; Cederberg et al., 2021). The so-called “long walks” provided the greatest concordance and least error, and this may be because many of the devices use the quasi-periodic nature of the acceleration signal during walking. This approach is most accurate when the rhythmicity of gait is high and variability is low. However, these same factors may adversely affect gait detection in PD when there is increased axial rigidity and loss of the normal rhythmicity of walking, and these same people may be less inclined to undertake “long walks.” Consequently, there may be some subjects who may be prone to higher step counting error rates when walking is measured for extended periods outside the laboratory.

The first aim of this study was to establish the algorithm’s step counting accuracy in normal walkers (with or without PD) and in people with an abnormal gait due to PD by comparing steps counted by an observer with the algorithm step count during videoed extended walking. The second aim was to use this information to establish whether the likelihood of a low rate of errors in step count could be predicted from data obtained from a wrist-worn sensor. The intention was to use the sensor data alone, without the need for confirmatory video to identify cases in whom the step count was reliable and not, in the first instance, to classify people as normal or abnormal walkers. The third aim was to examine the relationship between bradykinesia and step count in PwP whose risk of errors in step count was low.

### Method

To aid the reading of this study, a brief overview is provided here. First, in this section, the Parkinson’s KinetiGraph’s (PKG, Global Kinetics Corporation™, Australia), which is a system that uses data from a wrist-worn data logger, is described. Next, the PKG’s step detection system and manual step counts were compared in cohort 1 (48 PwP and 10 controls) while their walking was videoed (PKG_*video*_ v Manual_*step count*_). Examination of the spectral density and autocorrelogram indicated that accelerometry data recorded over 6 days could also be used to predict which subjects had low error rates (as determined by comparison between Manual_*step count*_ and PKG_*video*_) while the PKG recording is performed without any specific task-based requirement. The method for detecting this in the 6-day PKG (PKG_6__*day*_) is also described in this section, but the results of applying to PKGs from subjects in cohorts 2 and 3, consisting of 190 controls and 155 PwP, respectively, are described in the “Results” section.

### Ethics Approval

All cohort 1 participants gave written consent to participate, and approval for their study was provided by the St Vincent’s Health Hospital (Melbourne) Human Research and Ethics Committee. All participants in cohorts 2 and 3 provided written consent for their data to be used in future studies. All studies were carried out in accordance with the guidelines issued by the *National Health and Medical Research Council of Australia* for Ethical Conduct in Human Research (2007, and updated May 2015) and in accordance with the ethical standards laid down in the 1964 Declaration of Helsinki and its later amendments.

### The Parkinson’s KinetiGraph

**FIGURE 1:**
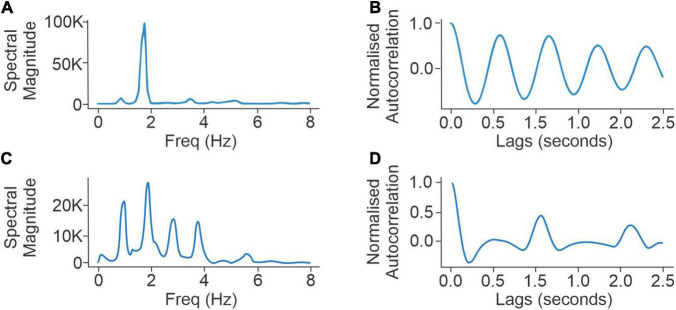
Panels **(A,B)** compare the spectral densities (*left*) and autocorrelation function (*right*) of a normal walker with low scores for UPDRS III questions for axial rigidity (UPDRS III 3a–3e = 2), postural stability (UPDRS III 3.10-12 = 1), UPDRS III (overall) (=20), and low FoG score (=5). Panels **(C,D)** compare the spectral densities (*left*) and autocorrelation function (*right*) of an abnormal walker with high responses to UPDRS III questions for axial rigidity (UPDRS III 3a–3e = 11) and postural stability (UPDRS III 3.10-12 = 4), UPDRS III (overall) (48), and high FoG score (=11).

The PKG system consists of a wrist-worn data logger, a series of algorithms that produce data points for bradykinesia and dyskinesia (Griffiths et al., 2012) every 2 min of recording and a report (or PKG), which plots these 2-min scores against the time of day (Figure 1; Griffiths et al., 2012).

The Bradykinesia Score (BKS) is produced by applying algorithms to the accelerometry data collected from each 2 min of recording (Griffiths et al., 2012). These BKS scores ranged from 0 to 140 with scores above 80 being associated with sleep. Thus, the scores relevant for this study were those less than 80. Data are typically collected for 6 days, and the median of BKS (mBKS) less than 80 between 09:00 and 18:00 was used in this study as a representation of the overall level of bradykinesia for that subject.

The PKG has a step count algorithm applied to the accelerometry data. Step count is estimated using the autocorrelation function of normalized triaxial acceleration signals. This procedure relies on the observation that during walking the autocorrelation function, which is the cross-correlation of a signal with its time-lagged replicas and is presented as a function of time lags, shows peaks at gait cycles (Moe-Nilssen and Helbostad, 2004), also known as dominant gait periods. In this study, the normalized acceleration at each time sample (sampling rate of 50 Hz) is calculated as the Euclidean norm of the acceleration samples corresponding to the *x*, *y*, and *z* axis (Mannini et al., 2013). The resulting one-dimensional signal is band-pass filtered to only include frequencies that are relevant to walking. To use the autocorrelation-based gait analysis technique, a 4-s neighboring window is used at each second to calculate an autocorrelation function (Moe-Nilssen and Helbostad, 2004). The gait period is defined as the lag to the second dominant peak (Figure 1B) in the resulting autocorrelation function (which is twice the step period).

### Accuracy of Parkinson’s KinetiGraph Step Count Compared to Steps Counted From a Video

Subjects in cohort 1 were videoed while walking and wearing a PKG (see below for video method). Cohort 1 consisted of 48 PwP and 10 people without PD (controls). Recruitment of PD subjects was explicitly directed at ensuring that a proportion of the PwP were normal walkers (*N* = 21) while PD clearly affected the gait of the remaining PwP (*N* = 27). Gait was assessed as “normal” or “abnormal” by a movement disorder specialist using factors covered by items 3.10–3.14 in the Movement Disorder Society Unified Parkinson’s Disease Rating Scale (MDS-UPDRS) Part III, but more explicitly, the following features. Subjects with normal gait (with or without PD) walked with symmetrical arm swing, with normal axial rotation of pelvis and shoulders, and with symmetrical foot strike. A blinded assessor could not confidently identify the presence of PD based only on an examination of the video. Any axial bradykinesia, postural asymmetry, or diminished arm swing should not have a greater effect on gait than common orthomechanical factors in the same age group. FOG questionnaire (FOG-Q) scores, performed in 38 PwP, were elevated in those with abnormal gait (9.5 ± 5.2 SD), but were low in those with normal gait (2.9 ± 3.6 SD; Table 1). In the 23 PwP who undertook an MDS-UPDRS III assessment, scores were also higher in the group with an abnormal gait (38 ± 11 SD) compared to those with a normal gait (28.1 ± 13.4 SD). Scores in MDS-UPDRS III questions 3.10, 3.11, and 3.12 tended to be higher when gait was abnormal (3.1 ± 2 SD) compared to normal walkers (1 ± 1 SD), in keeping with the basis for classification of an abnormal gait (Table 1). The use of a cane was not prohibited, provided it was not used in the arm that wore the PKG. Subjects who needed walkers were excluded. PwP were assessed on their usual dose of PD medications and in their “best” state. Almost all PwP (45/48) wore the PKG for 6 days (PKG_6 day_) within a few days of being videoed while walking. The mean age of controls was 69.9 (SD = 2.7), and the mean age of PwP was 71.6 (SD = 8.1).

**TABLE 1:** Description of cohorts.

Cohort	Control	PD	Description
1	10	38	Section 2.5. Recruited to ensure both normal walkers (*N* = 21) and PwP with clearly affected gait (*N* = 27)
2	190	-	Section 2.7. No history of PD or other neurodegenerative disorder. Aged 60 years or over (mean age of 70 (SD=6).
3	-	155	Section 2.7. Mean age 69 years (SD=5). Relevant clinical scales and demographic scores are in Table 4.

**TABLE 2:** Comparison of normal and abnormal walkers in cohort 1.

	Normal walkers mean (SD), [X]^Φ^	Abnormal walkers mean (SD), [X]Φ
Step count in video	396.95 (85.0) [21]	395.59 (87.0), [27]
Error rate	3.86 (3.1), [21]	11.42 (16.8), [27]
UPRDS III*	28.11 (13.4), [9]	38.0 (11.0), [14]
UPDRS III (3.10-12)*	1.0 (1.0), [9]	3.14 (2.0), [14]
UPDRS III Rigidity (3a–e)*	4.78 (3.1), [9]	5.29 (3.0), [14]
FOG score*	2.86 (3.6), [14]	9.54 (5.2), [24]

*For PwP only; ϕ number for whom assessment was available.

**TABLE 3:** Comparing the performance error prediction using spectral entropy features vs. number of autocorrelation peaks.

Cross validation (3-fold) performance metric	H_5%_
Specificity	0.79
Precision	0.88
Recall	0.79
F1	**0.84**
ROC-AUC	**0.76**
HPER (*E*_*total*_ ≥ 3%)	14
LPER (*E*_*total*_ < 3%)	29

*Measures in bold have significant p values.*

**TABLE 4:** Demographics for Cohort 3.

Measure	Category	No.	Mean	Std. dev	*p*-value Φ
Age	All	155	68.5	5.0	
	HPER	44	69.2	5.0	0.24
	LPER	111	68.2	4.8	
Gender (%F)	All	155	47%		
	HPER	44	52.3%		0.42 §
	LPER	111	45.05%		
Disease duration (years)	All	155	6.0	3.8	
	HPER	44	6.75	4.6	0.11
	LPER	111	5.65	3.4	
**Hoehn and Yahr**	**All**	**153**	**2.0**	**0.6**	
	**HPER**	**43**	**2.2**	**0.7**	0.001
	**LPER**	**110**	**1.9**	**0.5**	
MDS-UPDRS I	All	148	11.1	5.5	
	HPER	42	12.4	6.4	0.07
	LPER	106	10.56	4.9	
**MDS-UPDRS II**	**All**	**184**	**10.5**	**6.0**	
	**HPER**	**42**	**12.43**	**6.8**	0.01
	**LPER**	**106**	**9.7**	**5.5**	
**MDS-UPDRS III**	**All**	**154**	**35.6**	**10.5**	
	**HPER**	**44**	**40.9**	**10.7**	<0.0001
	**LPER**	**110**	**33.5**	**9.7**	
**MDS-UPDRS III Postural Stability Gait**	**All**	**154**	**2.4**	**1.6**	
	**HPER**	**44**	**2.9**	**1.8**	<0.01
	**LPER**	**110**	**2.2**	**1.5**	
**MDS-UPDRS III Q 10–14**	**All**	**154**	**4.1**	**2.8**	
	**HPER**	**44**	**4.8**	**3.2**	0.04
	**LPER**	**110**	**3.8**	**2.5**	
MDS-UPDRS IV	All	153	4.8	3.7	
	HPER	43	4.3	3.8	0.32
	LPER	110	5.0	3.7	
**MDS-UPDRS Total**	**All**	**147**	**62.3**	**17.7**	
	**HPER**	**41**	**71.0**	**19.4**	0.0002
	**LPER**	**106**	**59.0**	**15.8**	
**PDQ 39**	**All**	**150**	**28.3**	**18.1**	
	**HPER**	**43**	**34.8**	**21.3**	0.006
	**LPER**	**107**	**25.9**	**15.9**	
MoCA	All	155	26.2	2.3	
	HPER	44	26.1	2.5	0.6
	LPER	111	26.3	2.3	
NMS Quest	All	150	9.2	5.0	
	HPER	43	9.7	5.9	0.48
	LPER	107	9.0	4.5	

*Φp-Value of two-sided t-test comparing high and low error groups (HPER vs. LPER), except for gender (§ Chi-squared test). Measures with significant differences between HPER and LPER are bold.*

*Note that “n” represents the total number of subjects for whom values were available.*

#### Video Assessment of Gait

The PKG_*video*_ and the Manual_*step count*_ were obtained as follows. Subjects were directed to walk circuits of a 3-m-wide corridor between two points 30 m apart at their usual pace continuously for 4 min while being videoed and wearing a PKG that was synchronized with the video. The video steps were counted over the whole 4 min with a footfall from either foot being a step. A step count error rate was calculated:


$$E=100\times \frac{\left|S_{e\,s\,t}-S_{r\,e\,f}\right|}{S_{r\,e\,f}}$$

where *S*_*est*_ and *S*_*ref*_ are the number of steps estimated from the PKG_*video*_ and Manual_*step count*_, respectively. Step counts and error rates in controls and PwP classed as normal walkers were very similar. Therefore, in further analyses, controls and normal walking PwP were pooled as “normal walkers” and compared with abnormal walkers (Table 1).

### Error Prediction in PKG_6 day_

As the highest error between the walking algorithm and the step count from the video was in PwP who had an abnormal gait, it is plausible that these participants do not generate the strong and regularly spaced peaks in the autocorrelation of the accelerometry data that occurs in inherently rhythmic normal walking. These (harmonic) peaks are necessary for accurate gait detection by autocorrelation, so their attenuation may contribute to errors in gait detection. This view was supported by inspection of the spectrograms obtained from the accelerometry data recorded from PKG_*video*_. Figure 1 compares autocorrelation peaks (left: spectral, right: temporal) of an abnormal walker (PwP 1) with a “normal” walker (PwP 2) (Figure 1). As this loss of spectral harmonics accurately predicted that the algorithmic step count would have high errors with respect to the video step count, the full 6-day PKG (PKG_6 day_), recorded close in time to the PKG_*video*_ in 45 PwP from cohort 1, was examined for similar characteristics in the spectrogram.

Segments of accelerometry in which walking was detected were extracted from each PKG_6 day_, noting that a separate and independent walking detection was used to identify walking prior to feature extraction. Entropy calculated from the power spectral density of the autocorrelation function was derived from each 10-s interval (indexed *i*) of these segments using the following procedure (Shannon, 1949):


$$H^{i}=\sum_{k\in W}P_{k}^{i}\,\text{log}\,\left(P_{k}^{i}\right)$$

where $P_{k}^{i}$ represents the *k*th component of the power density function calculated for the *i*th 10-s interval. The left column in Figure 1 shows two examples of $P_{:}^{i}$. The acceptable frequency range *W* is chosen to only contain frequencies between 0.5 and 8 Hz to exclude DC components as well as noisy non-walking high-frequency components. Timestamps were calculated at each 10-s interval with 5-s overlaps. Spectral entropy has previously been used in characterizing signal disorganization in a wide range of applications, including speech (Shen et al., 1998), biomedical signal processing (Vakkuri et al., 2004; Viertio-Oja et al., 2004), and signal fault detection (Pan et al., 2009). The spectral entropy of sequential 10-s epochs of walking segments from the PKG_6 day_ was summarized in a density distribution. Various quartiles (percentiles) of the distribution were examined as predictors of the error rate in the step count with the 5th percentile (left tail) of the spectral entropy distribution providing the best separation of subjects with low and high error.


$$H_{5\%}=P_{5\,t\,h}\,\left({H^{i}|}_{i\in P\,K\,G}\right)$$

where the operator *P*_5*th*_(.) returns the 5th percentile of the distribution of all *H^i^* for a subject’s PKG recording during walking.

This concords with the intuitive interpretation that a normal gait pattern is likely to produce lower spectral entropy (Figure 1B), whereas steps are harder to detect when the power spectral density shows patterns of abnormal gait, and peaks are less prominent (Figure 1B). Based on the distribution of errors, a threshold of *E*_*total*_≥3% vs. *E*_*total*_ < 3% was used to separate high error subjects from low error subjects of cohort 1.

The performance obtained from the H_5%_ of walking from the PKG_6__*day*_ exceeded other spectral entropy features in terms of combined specificity and sensitivity in predicting error rate in the video study (Table 2). Therefore, for the purposes of this study, the *H*_*5%*_ feature was used as the sole predictor of step count error in PKG_6__*day*_. This allowed PKG_6__*day*_ to be separated according to whether their predicted error rate (PER) was low (LPER: corresponding to <3% error in PKG_*video*_ compared to Manual_*step count*_) or high (HPER: corresponding to ≥3% error in PKG_*video*_ compared to Manual_*step count*_). This analysis was conducted based on a blinded testing regime using subjects from cohort 1 with approximately 30% of the population used for testing.

### Prediction of Error Rates in PKG_6 day_ Recorded From Control and PwP Cohort

This error predictor was then applied to PKG_6__*day*_ recorded from people in cohorts 2 and 3 (described below) and the effect of PD on step count is described in the “Results” section. The two cohorts are described briefly here. Cohort 1 was described earlier in the “Accuracy of PKG step count compared to steps counted from a video” section.

**Cohort 2.** This cohort (*N* = 190) had no history of PD or other neurodegenerative disorders and was used as a control group for comparison with PD. They aged 60 years or over with a mean age of 70 (SD = 6). While none of the subjects used walking aids, no details were known of orthopedic disturbances or of other medical conditions and no relevant clinical scales such as the Montreal Cognitive Assessment (MoCA) were available. All wore the PKG for 6 days. They were recruited previously, and their data were held on a database.

**Cohort 3** (Table 3). This cohort (*N* = 155) included PwP who participated in a previous study examining the contribution of PKG information in clinical decision-making (Woodrow et al., 2020). All participants had a PKG and an MDS-UPDRS performed before and after changes in dopaminergic therapy. Most (148 subjects) also had the Parkinson’s Disease Questionnaire (PDQ39) and MoCA. None were receiving advanced therapies. Their mean age was 69 years (SD = 5), and relevant clinical scales and demographic scores are shown in Table 3.

For all three cohorts, the focus has been on reporting step count measures for triaxial acceleration. Of all participants, 14 subjects from cohort 2 did not have all three axes available, for which step count results were generated using a similar pipeline but with only the *x*-axis. Due to their limited number, this did not significantly impact results.

## Results

### Error Prediction Applied to PKG_6 day_ From Subjects in Cohorts 2 and 3

In cohort 2 (i.e., controls), 84% (160/190) had an LPER and will be referred to as C_*LPER*_ and 18% (30/190) had an HPER and will be referred to as C_*HPER*_. Note that criteria for being a control were absence of PD or known neurodegenerative disorder and age ≥ 60 years. Thus, orthopedic or other mechanical problems that might affect fluency of gait may be present and contribute to the HPER. The mean ages of the LPER group (70.3 ± 5.8 SD) and HPER group (71.5 ± 6.6 SD) were not significantly different and were similar to the age of the overall cohort (70.5 ± 6 SD).

A total of 72% of cohort 3 (111/155) had an LPER and will be referred to as PwP_*LPER*_ and 28% (44/155) had a HPER (PwP_*HPER*_). The clinical characteristics of PwP (cohort 3) and the PwP_*LPER*_ and PwP_*HPER*_ subcohorts are shown in Table 3. While the total cohort had relatively moderate PD with average disease duration of 6.1 years, H&Y of 2, PDQ 39 of 30, and MDS-UPDRS III of 36, the HPER cohort had significantly higher H&Y, MDS-UPRDS III, and Total and PDQ 39 (Table 3). The MDS-UPDRS III questions for posture, stability, and axial features and the sum of MDS-UPRDS III Q10-Q14 were also statistically worse in the HPER cohort.

### Step Count in People With Parkinson’s Disease Compared With Controls

**FIGURE 2:**
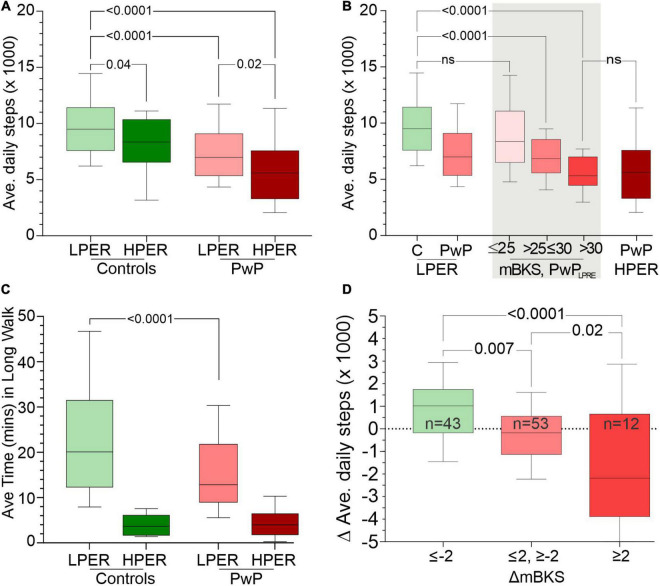
Panel **(A)** shows the average daily step count (*y-*axis) of C_*LPER*_, C_*HPER*_, PwP_*LPER*_, and PwP_*HPER*_ participants. Panel **(B)** compares the average daily step count (*y-*axis) of C_*LPER*,_ all PwP_*LPER*_, PwP_*LPER*_ stratified according to the PKG’s score for bradykinesia (mBKS) in the shade areas and PwP_*HPER*_. Note that the distribution of PwPLPRE with mBKS ≤ 25 has a similar distribution to that of C_*LPER*_, whereas those with the highest mBKS have a distribution similar to PwP_*HPER*_. Panel **(C)** shows the average time spent each day in long walks (walks > 1 min, *y-*axis) of C_*LPER*_, C_*HPER*_, PwP_*LPER*_, and PwP_*HPER*_ participants. Panel **(D)** shows the change (Δ) in average step count from before treatment to after treatment (*y-*axis: a positive number indicates an increase in step count). PwP_*LPRE*_ were sorted into three categories based on change (Δ) in mBKS from before treatment to after treatment where a negative (Δ) indicates an improvement in mBKS (and bradykinesia). All plots are box (median, 25th, 75th percentiles) and whiskers (10th and 90th percentiles) plots. The statistical differences between relevant plots are shown by *p*-values obtained from an ANOVA and Šídák’s multiple-comparisons *post hoc* test.

The average daily step count for controls and PwP with both LPER and HPER was plotted (Figure 2A) and shows that the average daily step counts of C_*LPER*_ are significantly higher than PwP_*LPER*_, with PwP_*LPER*_ on average taking 23% fewer steps. The average daily step counts of C_*HPER*_ and PwP_*HPER*_ were also plotted (Figure 2A) and their means were found to be significantly less than their respective counterparts whose PERs were low.

The relationship between bradykinesia and average daily step counts was assessed by comparing the median bradykinesia score (mBKS, one of the PKG’s measures of bradykinesia) and the average daily step counts of PwP_*LPER*_ (Figure 2B). The step count of PwP_*LPER*_ whose mBKS ≤ 25 (in the range of non-PD subjects) was similar to Control_*LPER*_ subjects. The mean MDS-UPDRS III and mean MDS-UPDRS Total of PwP whose mBKS ≤ 25 was 30.6 (SD = 10.0) and 55.9 (SD = 19.9), respectively, which is lower than the whole PwP_*LPER*_ cohort (Table 3). In contrast, subjects with a high mBKS had an average daily step count similar to PwP_*HPER*_ and similar MDS-UPDRS III (mean = 39.9, SD = 9.8) and MDS-UPDRS0 Total (mean = 67.3, SD = 13.4) (Table 4). Although the MDS-UPDRS III and MDS-UPDRS Total were higher in PwP with higher mBKS, the relationship to step count and these clinical scales was very weak. There was no relation between PDQ 39 and step count (data not shown).

Epochs of continuous steps were identified from PKG_6 day_ data and were separated into those fragments that lasted more than 1 min, known here as “long walks” (Figure 2C). Note that these were often contiguous with other epochs and resulted in walks much longer than 1 min. Control_*LPER*_ spent a median of 20.1 min/day in long walks compared to PwP_*LPER*_ (12.9 min). HPER participants spent far less time in long walks (3.7 and 4.0 min, controls and PwP, respectively). Total time walking was the sum of the time spent in long walks and the time spent in short walks (<1 min). Time spent in long walks expressed as a ratio of total time walking provides an indication of the proportion of time spent in long walks. This ratio was higher in Control_*LPER*_ (mean = 0.44, SD = 0.15) than Control_*HPER*_ (mean = 0.14, SD = 0.08). Similarly, the ratio was higher in PwP_*LPER*_ (mean = 0.39, SD = 0.16) than PwP_*HPER*_ (mean = 0.2, SD = 0.15). This implies that ∼40% of steps made by subjects with LPER are long walks, whereas only ∼14–20% of steps made by subjects with HPER are long walks.

The stride frequency of controls and PwP was also examined by calculating for each individual the median stride frequency of all steps taken over the 6 days of the PKG. The mean of these values for PwP_*LPER*_ (mean = 0.92 Hz, SD = 0.06) was slower (*p* = 0.045, *t*-test) than the mean of these means for Control_*LPER*_ subjects (mean = 0.94 Hz, SD = 0.06). Assessment of stride frequency in both controls and PwP with HPER was particularly affected by erroneous peak selection for autocorrelation, which makes stride frequency estimates unreliable in many instances.

### Effect of Treatment of Step Count

The original study of subjects in cohort 3 (Woodrow et al., 2020) was designed to examine the benefit of using sensor measurement and targets when treating PwP. Thus, the therapy of all 155 subjects was changed with the aim of optimizing their PD. This took between 2 and 6 months, and all participants had a PKG prior to changing therapy and when therapy was considered optimal (Woodrow et al., 2020). Thus, it was possible to examine 111 PwP_*LPER*_ and compare the change in the PKG’s bradykinesia score (mBKS) from first visit to last visit with the average daily step count from those same PKGs (Figure 1D). These cases were stratified into three groups, namely, those where there was a clinically meaningful change in mBKS (≤−2 mBKS final score minus first score) approximating to 5 UPDRS III points or more, those whose mBKS change little (±2 mBKS units), and those who deteriorated significantly ≥2 mBKS points).

There is a clear trend to an increase in steps taken by those whose mBKS improved and a decrease in steps taken by those whose mBKS deteriorated. This represents a 17% increase over the median daily steps of PwP_*LPER*_ (Figure 2D) when mBKS improved and 29% decrease from the median daily steps of PwP_*LPER*_ (Figure 2D) when mBKS deteriorated. The MDS-UPDRS III and Total scores were assessed around the same time as the “before” and “after” PKGs were performed. It is worth mentioning that there was no relationship between change in MDS-UPDRS scores and step count.

## Discussion

There has been interest in using ambulatory sensors to measure step count in PD since consumer grade devices providing this function first became available (Lamont et al., 2018; Straiton et al., 2018; Wendel et al., 2018; Lai et al., 2020; Svarre et al., 2020; Cederberg et al., 2021). While gait laboratories can provide detailed information about gait in PD, the hope has been that ambulatory measurement in an ecologically relevant setting might provide other information that cannot be gained from laboratory measurements. For example, step count may be an indirect marker of cognition, quality of life, and bradykinesia (Giladi and Hausdorff, 2006; Weiss et al., 2015; Kueper et al., 2017; Salazar et al., 2017; Yao et al., 2017), which are relevant to PD. Moreover, there is a research interest in the use of step count as an index of the severity and progression of PD. This study was a pilot aimed at understanding whether wrist-worn sensors can be used in all PwP or with a subset prone to a higher error rate in counting steps. A consequent question was whether subjects at risk of step count errors could be recognized from the accelerometry recording alone without first establishing accuracy by comparing with a video-assessed counts of steps.

There have been studies of step count accuracy of wrist-worn sensors in PD (Lamont et al., 2018; Straiton et al., 2018; Wendel et al., 2018; Lai et al., 2020; Svarre et al., 2020; Cederberg et al., 2021), but these regard PwP as a homogenous cohort with regard to risk of step count. However, this is unlikely because the well-known changes in walking and posture that occur as PD progresses are likely to lose the typical oscillatory energy produced by normal walking. Our assumption was that this group would likely present problems in step counting using the usual autocorrelation methods. Cohort 1 was explicitly selected to have both PwP who were normal walkers and PwP who walked with diminished axial rotation. The designation of participants as normal and abnormal walkers appears justified on the basis of their MDS-UPDRS III and FOG scores and the clear separation in error rates. Nevertheless, some “normal walkers” had high error rates. The most likely reason for this is that non-PD mechanisms may also contribute. In normal walking, the forward foot swing produces momentum that is transferred by axial rotation to the shoulder to produce the acceleration peaks measured in the arm. Accordingly, the mechanical effects of lumbar degenerative disease or spinal fusion surgery, which are common in the age group in this study, could impede the transfer of energy from foot to arm and thus account for a high error rate in a subject who did not have axial rigidity from PD. If step counting was the only aim, then placing a sensor on the pelvis or leg would overcome the problem of lumber rigidity but the simultaneously measured bradykinesia and dyskinesia obtained by wrist measurement using the PKG would be lost. Furthermore, we propose that the higher PER is an indirect measure of axial bradykinesia that cannot be as readily measured by sensors placed lower on the body.

The choice of H_5%_ in the accelerometry to predict error rate was informed by inspection of the spectral density obtained during videoed walking. This component provided good separation of the subjects in cohort 1 whose step count error rate had previously been established as high or low by comparison with videoed walking. As the aim was to select people with acceptable accuracy of step count, a boundary that favors a higher F1-score (balanced precision and recall) between LPER and HPER was chosen. A consequence of using the *H*_*5%*_ feature with a logistic regression is that it delivers a classification rather than a continuum of risk. However, a continuum would be desirable for the tracking of the course of disease by making repeated measures over time and observing an increasing risk of error until the boundary is exceeded. A larger cohort of videoed subjects with a richer feature set may be necessary to produce such a continuous scale of risk. While the effect of attenuation of the natural rhythmicity of walking on the autocorrelation function has been the main explanation for the increase in step count error, the marked reduction of time spent in long walks warrants discussion. The autocorrelation function searches for peaks within the next 4-s period. Thus, the step counter may be more error prone in detecting steps in walks of 5 s or less (∼4 or 5 steps). While it is plausible that PWP_*HPER*_ have less efficient walking and are thus inclined to avoid long walks, it is also plausible that when a large proportion of steps are in walks of less than 1 min (i.e., less than a long walking), there is also a higher chance that more steps will be in walks of 5 s or less and thus with more errors. It seems more likely that the fundamental problem is the loss of rhythmicity, but further investigation is needed to exclude the possibility that high proportion of steps in walks of 5 s or less are the cause of step count error.

The predicted risk of step count error was high (HPER) in 16% of the control population (cohort 2). These participants were controls in the sense that they did not have a neurodegenerative disorder, but they are not necessarily “healthy,” and it is to be expected that participants in both cohorts 2 and 3 will have the various musculoskeletal afflictions of people aged 60 or over. Furthermore, in the wider community, subjects with cognitive impairment and dementia walk more slowly than people with unimpaired cognition (Borges Sde et al., 2015) and slow walking speed and the extent of decline in walking speed bears some relationship to the risk of developing dementia (Beauchet et al., 2016; Quan et al., 2017; Hackett et al., 2018). While walking speed was not measured in this study, it is frequently associated with fewer steps.

Based on the incidence of HPER in the control population, it might be expected that 16% (∼25) of the 155 PwP in cohort 3 may have had non-PD factors contributing to a high predicted error, although this would not in itself explain the high UPDRS III Q10–14 scores, or the high FOG scores associated with PWP_*HPER*_. Tremor did not appear to contribute to erroneous step counting by providing spurious peaks for the autocorrelation function. PD tremor does produce resonant peaks in the spectrogram, but these are almost always above 4 Hz and above expected gait frequencies. However, the possibility that they may occasionally contribute cannot be excluded especially when tremor is lower in frequency and higher in energy such as with essential tremor. Dyskinesia increases spectral density in all frequencies above 3 Hz but is typically identified by the absence of peaks. It is thus unlikely that dyskinesia would produce spurious peaks, but the increase in energy across the spectrum may obscure walking generated peaks, making them difficult to identify. As reported in the results, neither tremor nor dyskinesia were overrepresented in PwP_*HPER*_ but future studies that systematically examine the effect of specific cases may reveal that in some instances both may interfere with the efficiency of the autocorrelation function.

Amongst PwP classified as a low risk of error (PwP_*LPER*_), there was a relationship between bradykinesia and step count (Figure 2B) and improvement in bradykinesia resulted in increase in average daily step count (Figure 2D). There was a modestly higher stride frequency in PwP compared with controls. Others have found a reduction in stride frequency and increasing bradykinesia (Chien et al., 2006; Nanhoe-Mahabier et al., 2011; Bryant et al., 2016; Orcioli-Silva et al., 2018; Del Din et al., 2019; Rehman et al., 2019; Corra et al., 2021). One reason for this difference with the published literature may be that the PwP in cohort 3 had relatively mild PD. The average MoCA and PDQ 39 was quite high, even in PwP_*HPER*_ and so a future study with a significant component of people with low MoCA and higher H&Y may be needed to reveal a lower gait frequency. Previous reports suggest that lower step count and slower stride frequency are driven largely by impaired executive function anxiety (Giladi and Hausdorff, 2006; Weiss et al., 2015; Kueper et al., 2017; Salazar et al., 2017; Yao et al., 2017). Another factor that may have contributed is that the second and subsequent peaks in the autocorrelation function were attenuated when the mBKS was higher. This may not have been enough to disturb the predicted error classification but may have interfered with estimation of stride frequency. It is not immediately apparent why the average daily step count has a relationship with mBKS but not with MDS-UPDRS III. Clearly the two measures encompass different aspects of bradykinesia with tremor, speech, and rigidity being factors included in the UPDRS but not in the mBKS. It might be expected that some of these would have associations (possibly inversely) with step count. The act of walking contributes to the spectral density in the accelerometry, and some of this is in frequencies that could contribute to the mBKS. However, the median time spent in walking long walks for PwP_*LPER*_ was 12.9 min, which represents only 4.8% of all the 2-min BKS that contributed to the mBKS. As the mBKS is a median value, it will be insensitive to an effect that influence only 5% of the scores. Even the PwP_*LPER*_ who walked the 90th percentile longest walk (30.3 min) walked for a small portion of the day.

The choice of the PER that separates LPER from HPER was based on cohort 1, which consisted of a greater range in severity of PD than cohort 3. To our knowledge, no participant in cohort 3 had FOG and participants had relatively mild disease (Table 3). A future study examining subjects with later stage disease and a greater proportion of subjects with clear axial involvement and FOG may help to clarify the choice of this transition point. The LPER/HPER classification is binary, whereas in reality the risk of error is a continuum from very low to very high. It might be expected that over time, particularly as factors that interfere with axial rotation become more intrusive, the PER progressively increases to the point that the classification change from LPER to HPER. Thus, there might be a period while the PER is very close to 3% that classification of LPER and HPER is unstable. Future studies are required to understand this better.

The average daily step count from the PKG for participants with a low PER is likely to be an accurate indication (within 3%) of the habitual number of steps taken by that individual over 6 days. However, the central tendency (mean or median) of the population (e.g., Control_*LPER*_ in Figure 2A) will also be accurate, with the error of 3% (along with the true biological variation) contributing to the variation. This is because there was no noticeable bias toward under- or over-count. In contrast, the average daily step count from the PKG for participants with a high PER will be less certain being greater than 3%. Even so, the central tendency of the HPER population (e.g., Control_*HPER*_ or PwP_*HPER*_ in Figure 2A) will still be an accurate indication for those populations because there was again only modest bias to undercount. However, the error rate will contribute more than 3% to the variation. The reason for making this point is that it is still possible to statistically compare step counts of high predicted error with a low predicted error (as in Figure 2A) using relevant statistical tests (e.g., ANOVA test). Thus, the 12% difference in the mean is an accurate representation of the difference in walking in these two cohorts. The important caveat is that it is far more difficult to be sure of the step count of an individual subject with a high PER.

The use of a step detection autocorrelation algorithm using data from a wrist-worn accelerometer appears to provide an accurate daily step count for assessing PD cohorts, although consideration should be given to the effect of increased variation associated with using PwP_*HPER*_. If the average daily step count of a specific individual is being considered, then the accuracy only meets the <3% error standard in PwP_*LPER*_. A larger cohort of more severe PD should be studied to fully understand the relationship between bradykinesia and step count and whether a transition from LPER to HPER occurs with the development of axial rigidity and risk of FOG. To achieve this, a different means of estimating risk of step count error may be required.

## Conclusion

Accelerometry data from wrist-worn sensors can be used to measure step count in PD with error rates of <3% providing that the rhythmicity of walking is near normal. This study used H_5%_ to detect loss of normal rhythmicity. PwP, whose step count error rates were high (according to this method), had more severe PD, and much lower step counts and time walking. Further studies of more severe PD are required to understand the development of a higher error rate and its association with axial rigidity and risk of FOG.

## Data Availability Statement

The raw data supporting the conclusions of this article will be made available by the authors on reasonable request.

## Ethics Statement

The studies involving human participants were reviewed and approved by St Vincent’s Health Hospital (Melbourne) Human Research and Ethics Committee. The patients/participants provided their written informed consent to participate in this study.

## Author Contributions

CF and SO contributed in coordinating subjects and video subjects while walking, arranging PKGs to be performed, and collecting data related to clinical scales. NS, HK, and MH were involved in analyses. MH wrote the manuscript. All authors reviewed the manuscript and approved the submitted version.

## Conflict of Interest

Global Kinetics Pty Ltd. (GK) is the manufacturer and distributor of the Parkinson’s KinetiGraph (PKG). NS was employed by GK. HK was employed by GK for most of the development of this manuscript. MH has equity in GK and has been CSO and acting CEO of GK. GK management had no role in the instigation, design, data collection, analyses, interpretation of data, or writing of the manuscript, nor have they been consulted as to whether the data should be published. The remaining authors declare that the research was conducted in the absence of any commercial or financial relationships that could be construed as a potential conflict of interest.

## Publisher’s Note

All claims expressed in this article are solely those of the authors and do not necessarily represent those of their affiliated organizations, or those of the publisher, the editors and the reviewers. Any product that may be evaluated in this article, or claim that may be made by its manufacturer, is not guaranteed or endorsed by the publisher.
